# Multicenter, Phase 1, Open Prospective Trial of Gastric Electrical Stimulation for the Treatment of Obesity: First-in-Human Results with a Novel Implantable System

**DOI:** 10.1007/s11695-020-04422-6

**Published:** 2020-03-04

**Authors:** G.F. Paulus, M. van Avesaat, S. van Rijn, A.M.E Alleleyn, J.M. Swain, T.L Abell, D.B. Williams, N.D. Bouvy, A.A.M. Masclee

**Affiliations:** 1 Department of General Surgery, NUTRIM, Maastricht University Medical Center, Maastricht, The Netherlands; 2 Department of General Surgery, Spaarne Gasthuis, Haarlem / Hoofddorp, Netherlands; 3 Division of Gastroenterology and Hepatology, Department of Internal Medicine, NUTRIM, Maastricht University Medical Center, Maastricht, The Netherlands; 4 HonorHealth Bariatric Center, Scottsdale, AZ USA; 5 Division of Gastroenterology, Hepatology, and Nutrition, University of Louisville, Louisville, KY USA; 6 Vanderbilt Center for Surgical Weight Loss, Vanderbilt University Medical Center, Nashville, TN USA

**Keywords:** Morbid obesity, Gastric electrical stimulation, Gastric emptying, Weight loss, Food intake, Quality of life

## Abstract

**Background and Aims:**

To assess safety of the Exilis™ gastric electrical stimulation (GES) system and to investigate whether the settings can be adjusted for comfortable chronic use in subjects with morbid obesity. Gastric emptying and motility and meal intake were evaluated.

**Method:**

In a multicenter, phase 1, open prospective cohort study, 20 morbidly obese subjects (17 female, mean BMI of 40.8 ± 0.7 kg/m^2^) were implanted with the Exilis™ system. Amplitude of the Exilis™ system was individually set during titration visits. Subjects underwent two blinded baseline test days (GES ON vs. OFF), after which long-term, monthly follow-up continued for up to 52 weeks.

**Results:**

The procedure was safe, and electrical stimulation was well tolerated and comfortable in all subjects. No significant differences in gastric emptying halftime (203 ± 16 vs. 212 ± 14 min, *p* > 0.05), food intake (713 ± 68 vs. 799 ± 69 kcal, *p* > 0.05), insulin AUC (2448 ± 347 vs. 2186 ± 204, *p* > 0.05), and glucose AUC (41 ± 2 vs.41 ± 2, *p* > 0.05) were found between GES ON and OFF. At week 4, 13, and 26, a significant (*p* < 0.01) reduction in weight loss was observed but not at week 52. At this time point, the mean excess weight loss (EWL) was 14.2 ± 4.5%.

**Conclusion:**

Gastric electrical stimulation with the Exilis™ system can be considered as safe. No significant effect on food intake, gastric emptying, or gastric motility was observed. The reduction in weight loss with Exilis™ GES was significant but short lasting. Further electrophysiological research is needed to gain more insight in optimal stimulation parameters and lead localization.

## Introduction

Bariatric surgery is the only long-term effective treatment for morbid obesity. However, only a small percentage of potentially eligible subjects will ever undergo a bariatric procedure [1]. Bariatric surgical procedures such as laparoscopic adjustable gastric banding (LAGB), laparoscopic sleeve gastrectomy (LSG), and Roux-en-Y gastric bypass (RYGB) [2, 3] modify gastrointestinal anatomy and physiology, require lifelong medical surveillance, and are associated with a considerable amount of complications and long-term adverse effects such as GERD, chronic vomiting, dumping syndrome, and nutritional deficiencies. Taking the abovementioned considerations into account, there is room for other, less invasive therapies for morbid obesity. In this respect gastric electrical stimulation (GES) has been studied for over a decade as a minimally invasive, anatomy-preserving alternative for traditional bariatric procedures for the management of morbid obesity [4, 5]. The technique aims to impair gastric motor function and to modulate afferent signaling from the stomach, leading to delayed gastric empting with prolonged gastric distension and enhanced satiety, thus resulting in decreased food intake and weight loss [6].

Initial results with the Transcend Implantable Gastric Stimulator (IGS) were promising, but consecutive double-blind randomized controlled trials initiated between 2000 and 2005 failed to show a clearly beneficial effect on body weight relative to sham-stimulated controls [7–9].

Up to now, in the reported clinical trials, only a narrow range of stimulation parameters and electrode configurations have been evaluated. Most clinical data on GES for obesity have been obtained using a single pulse frequency and duty cycle setting (40 Hz, 2 s On-3 s Off). Unfortunately, the efficacy and functional implications of these settings have not been systematically explored, neither in animals nor in humans. Nearly all subjects in these prior GES clinical trials were implanted with a single model of bipolar intramuscular lead, embedded in the stomach wall near the middle of the lesser curvature.

In a 5-year period of extensive animal studies in rodents, canine, and swine, each major component of GES was systematically reexamined. It was shown that a pulse width of > 2.0 ms [10], a 40 Hz pulse frequency, continuous stimulation (16 h On-8 h Off), and pre-pyloric pulse delivery led to an optimal delay in gastric emptying, gastric distension, and reduction in food intake [10]. Moreover, chronic, daily delivery of the GES treatment resulted in weight loss. The encouraging results of these animal studies have been used to define the required capabilities of the current Exilis™ system.

The aim of this early feasibility study was to gain first-in-human experience, to assess safety of the GES system, and to investigate whether the settings can be adjusted for comfortable chronic use. Furthermore, we aimed to discover whether acute gastrointestinal (GI) and feeding effects, as observed in animals, could be reproduced in humans. In addition, we aimed to enhance understanding of the mechanisms of action by which GES induces weight loss.

We hypothesized that GES for obesity would be safe, decrease food intake, and induce weight loss, possibly through a delay in gastric emptying.

## Methods

### Study Design

We initiated a multicenter, phase 1, open prospective cohort study conducted in the Netherlands and the USA. The study was approved by the medical ethics committee of all participating hospitals and was conducted in full accordance with the Declaration of Helsinki (latest amendment by the World Medical Association in 2013). Participants gave written informed consent prior to participation. This study was registered in the US National Library of Medicine (http://www.clinicaltrials.gov, NCT01823705).

### Subjects

Patients were enrolled via the outpatient clinics of the participating hospitals. Patients were considered eligible to enroll in this study if they were weight stable, between 21 and 64 years of age and had a body mass index (BMI) of 40–45 kg/m^2^ or 35–39.9 kg/m^2^ with at least one weight-related comorbidity (e.g., nonalcoholic steatohepatitis, hypertension, dyslipidemia, obstructive sleep apnea, arthrosis). In case a subject was diagnosed with diabetes mellitus, the diagnosis had to be made within the last 7 years, had to be currently treated with oral agents only, and had to have an HbA1c ≤ 8%. Exclusion criteria were prior major GI surgery (including bariatric surgery), pregnancy or the intention to become pregnant, functional and/or motility disorders, and medical, surgical, or psychiatric conditions that would limit study participation. Possible candidates underwent evaluation by a psychologist and a dietician before they were included in the trial. They were excluded from participation if behavioral issues (personality disorder, depression, and/or binge eating) were observed.

### Procedure

**Fig. 1:**
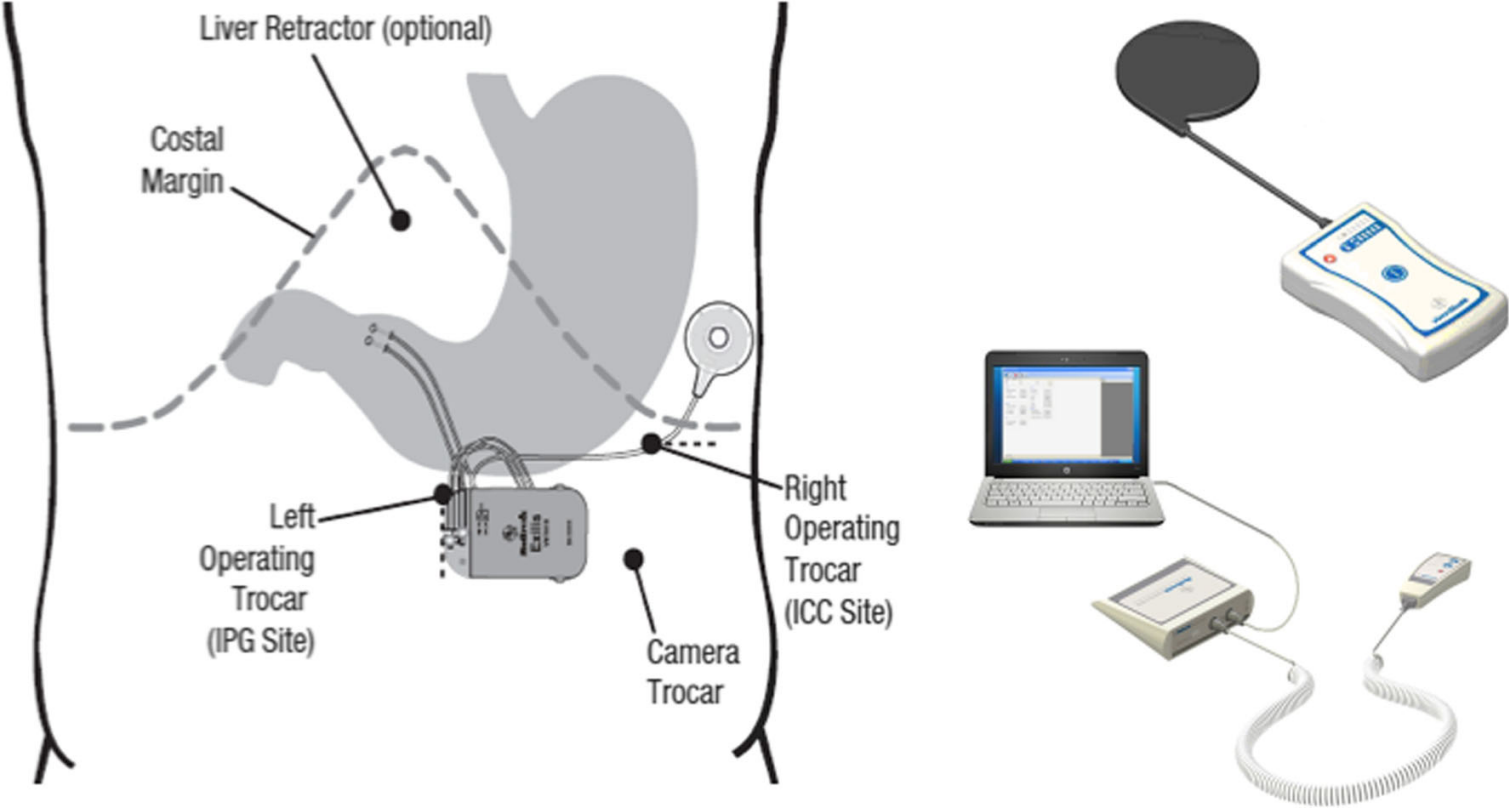
Implanted components (left), patient charging system (upper right), and external programming interface (lower right)

The system was implanted under general anesthesia. The implanted components (Fig. 1) consisted of a pulse generator (IPG, Model VNT0016 Version 3, Medtronic, Minneapolis, USA) with implantable charge coil (ICC, Version 1, Medtronic, Minneapolis, USA) and two 35 cm insulated unipolar leads (Model 4351 M, Medtronic, Minneapolis, USA). The leads were laparoscopically implanted into the muscle wall of the gastric antrum and were placed 3 to 5 cm proximal to the pylorus and parallel to the lesser curvature. A fixation disk was used to suture the leads to the serosal surface of the stomach. During placement of the leads, upper endoscopy was performed to prevent intraluminal placement of the electrodes. If indicated, the leads were reinserted. When correct placement of the leads was confirmed, the distal ends were pulled through the skin incision of the caudal trocar and connected to the IPG. The IPG was implanted in a subcutaneous pocket (1.5 to 4.5 cm deep) off midline between the patient’s iliac crest and ribs and sutured in place. A similar pocket for the ICC was created above the ribs in the subcostal region of the 9th rib along the anterior axillary line. The ICC receives electromagnetic energy through magnetic coupling with the external charge coil to recharge the IPG (Fig. 1). ICC and leads were connected to the IPG, checked for integrity, and switched off at the end of the procedure with the programming interface (Fig. 1). The final position of the entire Exilis™ system was recorded with a postoperative abdominal X-ray. The implant surgery was followed by a 2-week recovery period prior to continuation of the study protocol.

### Study Protocol

**Fig. 2:**
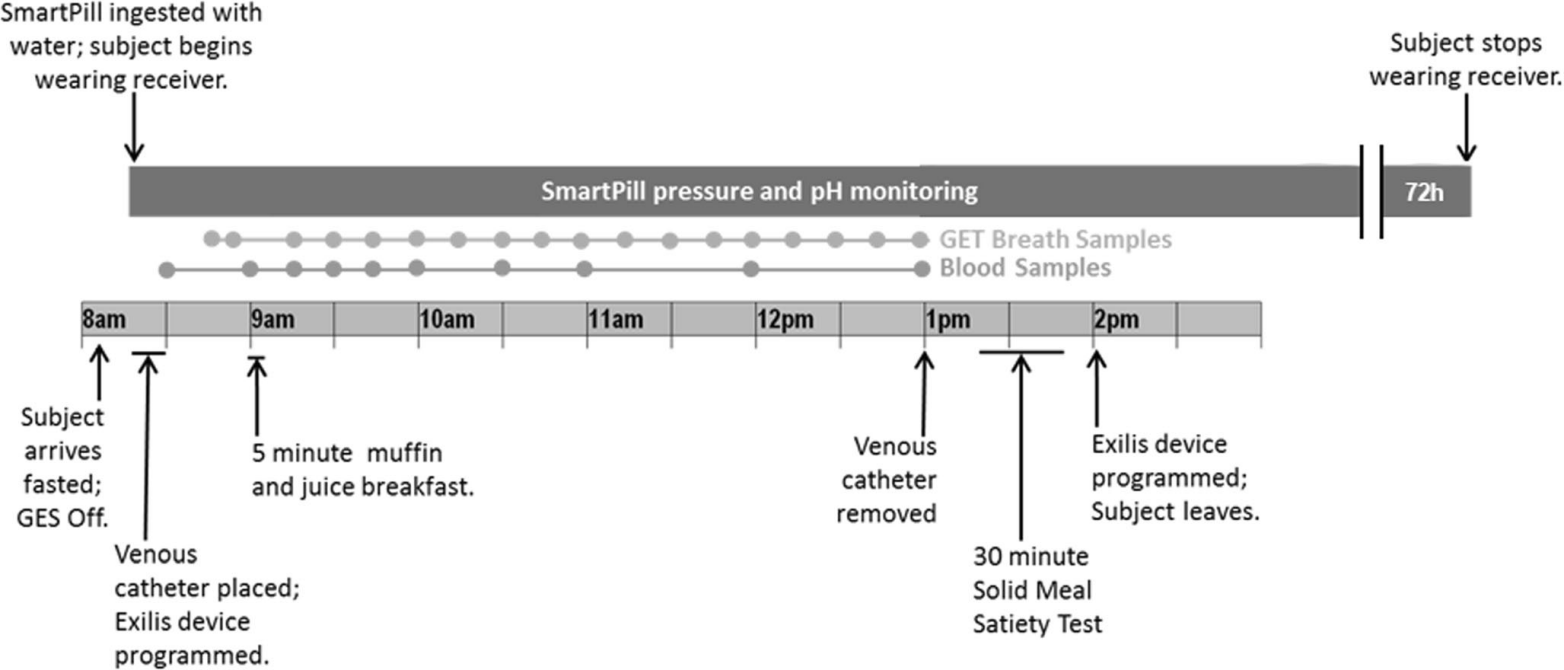
GI function testing

The study protocol continued with four amplitude titration visits (visits A, B, C, and D) occurring at weekly intervals. During the first of these visits, the IPG was switched on, and subjects underwent sensory threshold tests in which they were exposed to stimulation at progressively higher amplitudes. Visit A was used to identify the lowest amplitude that caused any visceral sensation, while at the fourth and final titration visit, subjects were programmed to the highest comfortable pulse amplitude. Fixed parameters of the IPG were a pulse width of 5.0 ms, frequency of 40 Hz, and a continuous duty cycle for 16 h per day (off during 8 h at night). The amplitude titration visits were followed by two GI function test days performed in randomly assigned order and repeated twice (once with GES ON and once with GES OFF). Each GI function test day was preceded by a washout period (GES OFF) of 7 days, and subjects were blinded to the assigned GES treatment. Testing included simultaneous measurement of gastric emptying (using a stable isotope breath test), gastric motility (SmartPill®), plasma concentrations of glucose and insulin, and food intake over a 4-h period in the morning following an overnight fast (Fig. 2). After completion of the GI function test days, each participant was programmed to GES ON (with the amplitude as determined during titration visit D), and long-term follow-up was started. Participants had to charge the IPG (by connecting the external charge coil to the ICC) once every 48 h. Monthly follow-up visits were planned during the first 12 months. Furthermore, GI function tests (with GES ON) were scheduled at weeks 26 and 52 of follow-up.

### GI Function Tests

Participants arrived at the hospital after an overnight fast. After programming of the GES device into ON or OFF mode (randomly assigned), baseline blood and breath samples were collected. In order to measure GI function, participants swallowed a SmartPill® (Buffalo, NY) and ingested a standardized breakfast muffin mixed with ^13^C-octanoic acid and 200 mL of orange juice (82 kcal, 19.2 g sugar). Blood and breath samples were collected at regular intervals during a 4-h period. Test days ended with the ingestion of an ad libitum pasta meal (in the Netherlands: Lasagna Bolognese; Plus Supermarket; energy density per 100 g: 160 kcal, 11 g carbohydrates, 7.1 g protein, and 9.4 g fat; in the USA: Macaroni and Cheese; Stouffer’s; energy density per 100 g: 142 kcal, 15 g carbohydrates, 6 g protein, and 6 g fat), which participants could eat until comfortably full.

### Gastric Emptying

Gastric emptying was determined by using the gastric emptying breath test kit provided by Metabolic Solutions, Inc. (Nashua, NH). As mentioned above, 100 mg of ^13^C-octanoic acid was mixed into the standardized breakfast muffin (350 kcal, 64 g carbohydrates, 9 g protein, 7 g fat) ingested at *t* = 0. Breath samples of ^13^CO_2_ were obtained twice at baseline and every 15 min for 4 h following ingestion of the breakfast meal. Samples were analyzed using a gas isotope ratio mass spectrometer, and gastric emptying halftime and lag time were calculated using the Ghoos model [11].

### SmartPill®

A wireless motility capsule (WMC, SmartPill®, Buffalo, USA) was used to obtain pressure data of the stomach and small intestine. The WMC has several sensors that monitor pH, pressure, and temperature and transmits these data to a receiver. Our participants swallowed the SmartPill® after consuming breakfast (breakfast muffin with orange juice) at each GI function test. Subjects wore the data receiver to enable continued data collection from the capsule for 72 h (or until the capsule was passed during a bowel movement). The motility index was calculated as follows: Ln(sum of pressure amplitudes × number of contractions (Ct) + 1) (from Camilleri et al., 1985) [12].

### Blood Samples (Glucose and Insulin)

Sodium fluoride and SST II Plus gold tubes (Becton & Dickinson, New Jersey, USA) were used for determination of serum glucose and insulin, respectively. Glucose measurements were performed on an Olympus AU 640/2700/5400 (Olympus, Tokyo, Japan). SST II Plus gold tubes were stored at room temperature for 30 min before centrifugation at 3000 rpm, 20 °C for 15 min. Serum insulin was measured using the Linco Human Insulin-specific RIA (HI-14 K) on a gamma counter with an inter-assay precision of 2.9–6.0%.

### Quality of Life Questionnaires

The Impact of Weight on Quality of Life-Lite (IWQOL-Lite) and the Multi-purpose Short Form Survey-12 (SF-12) were used to measure quality of life. Both surveys were administered at screening visit, week 0, 13, 26, and 52 postoperatively. The SF-12 health survey consists of 12 questions extracted from the SF-36 survey. It includes both a physical (PCS) and mental component score (MCS). A greater score indicates generally better health [13]. The IWQOL-Lite consists of 31 questions extracted from the longer IWQOL (74 questions). An increase of 7–12 points shows a meaningful improvement in quality of life [14].

### Statistical Analysis

Statistical analyses were performed using SPSS 23.0 (IBM Corporation, Somers, NY). Data were visually checked for normality and for constant variance of residuals by plots of residuals vs. corresponding predicted values. If data were not normally distributed, log transformation was applied for further analysis of the data. Area under the curve (AUC) was calculated by the trapezoid rule. All variables were compared with a mixed analysis of variance model that included the fixed factor test day and random factor subject. For insulin and glucose (multiple time points per test day), time and the interaction between test day and time were added to the model. If a statistically significant intervention effect occurred, a post hoc Bonferroni test was performed. Data are presented as the mean ± SEM (unless specified otherwise) and considered significant at *p* < 0.05.

## Results

### Participants

After screening 32 subjects, 12 were excluded for failure to meet inclusion criteria. A total of 20 subjects (3 male and 17 female with a mean age of 43.6 ± 1.6 years., a mean weight of 116.4 ± 4.1 kg, and a mean BMI of 40.8 ± 0.7 kg/m^2^) were included after giving informed consent and were implanted with the Exilis™ system. Considering comorbidities, one subject had diabetes mellitus, two had dyslipidemia, and five hypertension. The procedure was performed without any serious adverse events in all 20 subjects. All patients were discharged after one night, and none of the patients had to be readmitted. With the exception of incisional hernias which had to be corrected surgically (*N* = 2), all other adverse events were mild and could be treated conservatively or with medication therapy (Table 1). At 26-week follow-up, 3 subjects had withdrawn from the study due to not reaching the desired effect (*N* = 17 remaining). At 52 weeks follow-up, another 4 subjects had withdrawn for similar reasons (*N* = 13 remaining). Most of the patients that withdrew from the study had a surgical revision to RYGB or LSG. They were therefore not included in further analysis.

**Table 1:** Adverse events

Adverse event	Action undertaken	N (%)
Misplacement of leads (inside stomach lumen)	Replacement of leads	2 (10%)
Liver laceration	Electrocautery	1 (5%)
Seroma at IPG site	None	3 (15%)
Wound infection at IPG site (superficial)	Antibiotic therapy	3 (15%)
Incisional hernia	Surgically corrected	2 (10%)

### Amplitude Titration Visits

All 20 subjects were able to undergo the amplitude titration visits at the desired time. At the first titration visit (A), intended to determine the lowest amplitude that caused visceral sensations, 60% of the patients were set on an amplitude of ≤ 8.5 mA. Eighty percent of subjects reached the maximum amplitude of 10 mA after titration visit two (B). Long-term follow-up started with 90% of subjects set to an amplitude of 10 mA (Table 2, amplitude settings).

**Table 2:** Amplitude titration visits

Amplitude	A	B	C	D
≤ 5	2 (10%)	1 (5%)		
6	1 (5%)		1 (5%)	1 (5%)
7	3 (15%)			
8	2 (10%)			
8.5	4 (20%)	2 (10%)		
9	1 (5%)	1 (5%)	1 (5%)	1 (5%)
9.5	1 (5%)			
10	6 (30%)	16 (80%)	18 (90%)	18 (90%)

Amplitudes after titration visit A, B, C, and D

### GI Function Tests

**Fig. 3:**
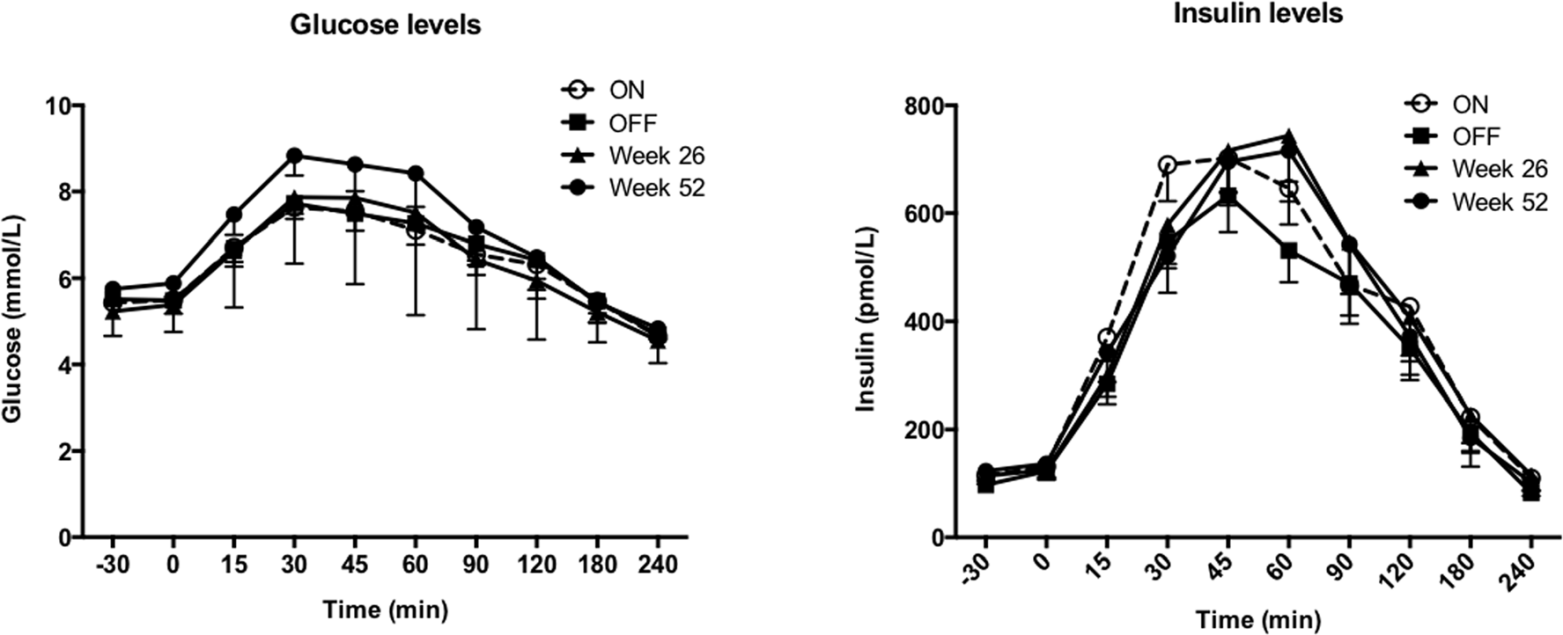
Plasma glucose (left) and insulin (right) concentrations over time (mean ± SEM) on the four different test days with GES ON, OFF, at week 26 and at week 52. No significant differences were observed

Ingestion of the breakfast muffin meal caused increases in plasma glucose and insulin concentrations that were not significantly different between the 4 test days (Fig. 3). Also, the areas under the curve (AUC) for glucose or insulin levels were not different between the 4 test days (Table 3). Gastric emptying halftime was not significantly different between GES ON versus OFF (202.9 ± 15.7 min versus 212.2 ± 13.6 min, respectively). At week 26, gastric emptying halftime was 191.6 ± 14.8 min, and at week 52, 161.6 ± 6.6 min, *p* = 0.07. Food intake was not significantly different between GES ON versus OFF nor was food intake at week 52 significantly different from baseline (Table 3). The GI motility index calculated from the SmartPill® recording was not significantly different between GES ON versus OFF.

**Table 3:** Results of GI function test (with follow-up)

	Screening	ON	OFF	Wk 26	Wk 52	P
Insulin AUC (mmol/l.min)		2448 ± 347	2186 ± 204	2187 ± 301	2388 ± 278	0.47
Glucose AUC (mmol/l.min)		41.4 ± 1.5	41.4 ± 2.0	41.0 ± 1.5	43.3 ± 2.6	0.60
GE T1/2 (min)	179.2 ± 8.3	202.9 ± 15.7	212.2 ± 13.6	191.6 ± 14.8	161.6 ± 6.6	0.07
Food intake (kcal)		712.7 ± 68.4	798.8 ± 68.9		800.2 ± 86.3	0.62
Motility index		53.4 ± 9.4	60.9 ± 10.5			0.60

All data are presented as mean ± SEM. *P* values are for test day effects determined by mixed analysis of variance model. AUC: area under the curve, GE T1/2: gastric emptying half time, ON: GI function test with GES ON; OFF: GI function test with GES OFF, Wk 26: week 26, Wk 52: week 52

### Weight Follow-up

**Fig. 4:**
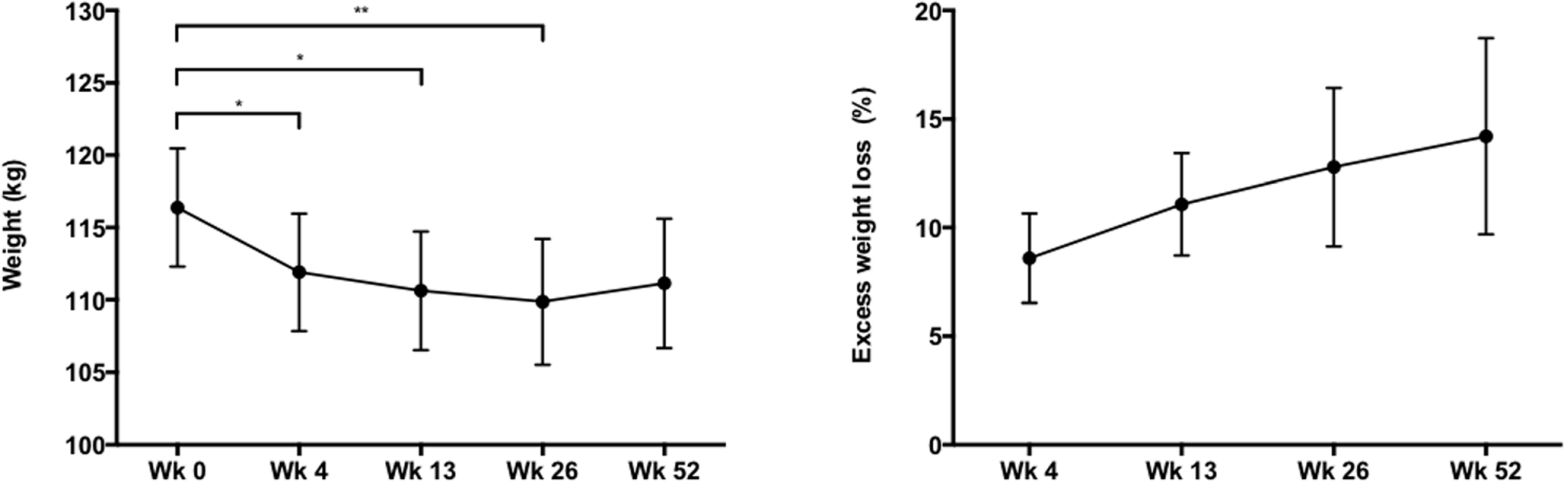
Weight (kg) and excess weight loss (%) at Wk 0 (*N* = 20), Wk 4 (N = 20), Wk 13 (N = 20), Wk 26 (*N* = 17), and Wk 52 (*N* = 13). ^*^p < 0.005. ^**^p < 0.01

Mean body weight at baseline was 116.4 ± 4.1 kg and decreased significantly to 109.9 ± 4.3 kg at week 26 (*p* < 0.01) as shown in Fig. 4. At week 52, body weight was not significantly different from baseline. The mean percentage of excess weight loss (%EWL) at 4, 13, 25, and 52 weeks was 8.6 ± 2.1%, 11.1 ± 2.4%, 12.8 ± 3.7%, and 14.2 ± 4.5%, respectively (Fig. 4).

### Changes in Systemic Parameters and Quality of Life (QOL)

No significant differences were observed in cholesterol levels, fasting glucose, HbA1c, and waist or hip circumference during the 1-year follow-up of this study (Table 4). Regarding QOL, significant differences were observed in SF-12 PCS and IWQOL-Lite total score. Mean SF-12 PCS improved from 41.3 ± 1.9 at screening to 46.6 ± 1.9 at 1-year follow-up (*p* < 0.05). Mean IWQOL-Lite total score improved from 55.4 ± 3.8 at screening to 75.0 ± 3.4 at 1-year follow-up (*p* < 0.001).

**Table 4:** Cholesterol, glucose, HbA1c, waist and hip circumference, and SF-12 and IWQOL-Lite outcome

	Screening	Wk 0	Wk 13	Wk 26	Wk 52	p
Triglycerides	1.33 ± 0.13	1.26 ± 0.16	1.40 ± 0.20	1.22 ± 0.14	1.38 ± 0.16	0.75
HDL	1.20 ± 0.07	1.23 ± 0.09	1.24 ± 0.09	1.33 ± 0.10	1.34 ± 0.10	0.08
LDL	3.04 ± 0.19	3.13 ± 0.19	3.04 ± 0.16	3.03 ± 0.18	3.21 ± 0.20	0.85
Total cholesterol	4.84 ± 0.22	4.88 ± 0.24	4.88 ± 0.22	4.88 ± 0.21	5.10 ± 0.27	0.75
Fasting glucose	5.54 ± 0.19	5.45 ± 0.16	5.64 ± 0.18	5.42 ± 0.19	5.45 ± 0.20	0.33
HbA1c	5.60 ± 0.10	5.43 ± 0.07	5.48 ± 0.07	5.45 ± 0.07	5.50 ± 0.11	0.35
Waist circumference	122.4 ± 3.2	114.1 ± 5.1	115.9 ± 3.0	114.6 ± 3.4	118.7 ± 3.2	0.08
Hip circumference	131.3 ± 1.9	122.9 ± 4.8	125.5 ± 1.9	125.8 ± 2.2	127.8 ± 1.7	0.1
SF-12 PCS	41.3 ± 1.9	44.4 ± 2.0	46.6 ± 1.7*	45.5 ± 1.7	46.6 ± 1.9*	< 0.001
SF-12 MCS	53.5 ± 2.8	56.4 ± 2.7	55.7 ± 2.4	58.4 ± 1.8	56.6 ± 2.7	0.40
IWQOL-Lite total score	55.4 ± 3.8	67.8 ± 3.7**	70.8 ± 3.4**	74.0 ± 3.7**	75.0 ± 3.4**	< 0.001

All data are presented as mean ± SEM. *P* values are for test day effects determined by mixed analysis of variance model. Significant differences were determined by using post hoc comparisons with Bonferroni’s correction. Wk 0: week 0, Wk 13: week 13, Wk 26: week 26, Wk 52: week 52

*Significantly different from screening, *p* < 0.05

88 significantly different from screening, *p* < 0.001

## Discussion

In this first-in-human study with the Exilis™ GES system, we have shown that the system can be used safely and that GES was induced without causing discomfort in any participant. Despite the absence of discomfort, subjects were able to accurately predict whether the pulse generator was turned on or off. At baseline, food intake and satiety were not significantly different between GES ON versus OFF. A significant reduction in body weight occurred until week 26. We observed an excess weight loss of 14% at 52 weeks. This percentage is comparable with data from studies of subjects on diet and/or exercise alone, but this effect should be considered as disappointing when compared to minimal invasive procedures, such as gastric banding (50%) or endoscopic gastroplication (35%) [3, 15]. Despite moderate weight loss, ad libitum food intake did not differ statistically significantly between follow-up moments. Furthermore, we did not observe changes in plasma glucose and insulin levels, while some other bariatric procedures are known to improve glucose metabolism, independent from weight loss [16].

Up to now various devices with different patterns of electrical stimulation have been evaluated for the treatment of morbid obesity. The first open and uncontrolled clinical trials investigating the Transcend® Implantable Gastric Stimulator (Medtronic Inc.) reported excess weight loss (EWL) varying in the range of 20–30% after 29 months of stimulation [17]. In the present study, we found a mean EWL of 14% at 52 weeks (corresponding with a mean weight loss of 6.5 kg). Our results are comparable to those found in the SHAPE trial investigating the Transcend® device and the studies using the Tantalus gastric electrical stimulatory device (DIAMOND™) [9, 18, 19]. Interestingly, an open, uncontrolled study using the closed-loop gastric electrical stimulation system Abiliti® showed a mean EWL of 29% at 12 months [15]. Although considerable variability in weight loss has been observed in several clinical studies, one could argue whether the abovementioned variations are related to differences in stimulation parameters, to anatomical localization of leads, to differences in stimulation paradigm, or to other factors. Extensive animal work preceding the present study focused on acquiring the most effective lead position and stimulation parameters. Based on the canine data, the Exilis™ system uses continuous (16 h per day), current-controlled, monophasic pulses (width of 5.0 ms) with alternating polarities and a fixed frequency of 40 Hz. Although an infinite variation in programmed parameters can be obtained, such as pulse width, frequency, and amplitude, it is evident that the total amount of energy delivered is of utmost importance. Yoa et al. expressed the energy delivered by GES in smA^2^ and found in humans that the stimulation energy required for the first visceral sensation varied between 112.5 and 480 smA^2^, while the highest tolerated stimulation energy varied between 480 and 3840 smA^2^ [20]. These authors also found that the subjects who were most sensitive to GES showed the greatest response to stimulation at 112.5 smA^2^, leading to a significantly decreased water intake and gastric emptying rate. The stimulation energy delivered by the Exilis™ pulse generator at the maximum amplitude of 10 mA is 1200 smA^2^. Although this energy level is well within the viscerally sensible and therapeutic range, no changes in food intake or gastric emptying rate were observed in the present study. An explanation for this lack of effect despite adequate stimulation is not readily available. It is possible that the lead position or specific stimulation parameters did not deliver the same amount of energy through the gastric tissue. Although the leads were placed 1 cm apart in both procedures, we placed them through the serosa of the stomach, while Yoa et al. used a transoral technique and placed them through the mucosa. Furthermore, our subjects may have been less sensitive to GES. The finding that a relatively large amount of energy was needed for visceral sensation supports this assumption.

Previous human in vivo experiments have shown a correlation between visceral sensitivity to GES and gastric responses, such as gastric motility and food intake. A higher response was noted in subjects more sensitive to GES [20]. A total of 30% of our subjects were titrated up to the maximum of 10.0 mA already at the first visit, before any visceral sensation had occurred (a higher setting was technically not possible). The amplitude that caused the first sensation varied greatly in the remaining subjects (Table 3), but in those who were more sensitive to GES, a greater clinical effect was not observed. Eventually, 90% of our subjects received chronic GES therapy at the highest technically feasible amplitude of 10 mA. The gradual increase in amplitude setting showed that subjects had milder visceral sensations at the fourth titration visit when compared to the first visit. This observation points to adaptation to GES after prolonged application and is in line with our findings that GES effects were most pronounced in the short postoperative period after implantation.

In the current study, we applied continuous (16 h a day) electrical stimulation with a standardized pulse width. In such a setting, adaptation to the signal may have occurred, eventually even resulting in loss of efficacy. Adaptation of the gastric smooth muscle to chronic GES for the treatment of obesity has been shown previously in a dog model [21]. Pulses with higher amplitude (i.e., higher stimulation energy) or pulse sequences that are unlikely to induce adaptation might be necessary in order to achieve adequate and long-term gastric responses. This supposition might explain why a GES system, with less frequent meal initiated stimulation results in a greater and more persistent effect on food intake and weight loss [22].

In the present study, we report several (minor) adverse events. Most adverse events were related to the IPG pocket (seroma, infection, hernia) and are most likely due to the relatively superficial placement of the IPG in the loose subcutaneous tissue. Ideally, the IPG would be sutured to the abdominal fascia, which could, however, cause connectivity problems in patients with a significant amount of subcutaneous fat. There were 10% incisional hernias that required correction. To reduce this percentage, more care should be taken to close the fascia around the leads or to tunnel the leads through the rectus abdominis muscle. Contrary to the popular bariatric procedures, a clear advantage of the current technique is its reversibility: all devices can be explanted without interference with GI anatomy and function.

Our study has several limitations. Due to the aims and deliverables of this study – to assess safety and preliminary effectiveness of the Exilis™ system – a control group was not included. In all studies and trials on interventions for weight loss, a control group is required to fully assess efficacy. Several studies have shown between 12 and 14% of excess weight loss in the control groups, which is comparable with what we observed with the present study [9, 23]. Therefore, we conclude that the additional effect of GES with the Exilis™ system with its current settings is limited. Up to now, substantial work with GES for the treatment of obesity has been performed, and results vary considerably. More essential basic research has to be performed before we come to clinical applications. Pacing protocols should be optimized to achieve physiologically and clinically useful outcomes. Essential electrophysiological knowledge of the human stomach is still lacking, and more basic electrophysiological research work should be done before proceeding to new pacing protocols [24]. Potentially, high resolution mapping of gastric slow-wave activity and the effects of gastric pacing on these waves may be a method to assess whether pacing protocols will be effective [25]. When optimal stimulation parameters have been assessed, we recommend that they will be tested in a blinded randomized placebo-controlled trial.

In conclusion, gastric electrical stimulation with the Exilis™ system can be considered as safe in humans. No significant effect on food intake, gastric emptying, or gastric motility was observed. The reduction in weight loss with Exilis™ GES was significant but short lasting. More basic electrophysiological research is needed to develop optimal GES paradigms.
